# Wet Surface Treatment for Preventing Pattern Collapse of Dry-Film Photoresist in Panel-Level Redistribution Layer Interposer Fabrication

**DOI:** 10.3390/polym18182249

**Published:** 2026-09-15

**Authors:** Seonwoo Kim, Suin Chae, Soobin Park, Hyunjin Nam, Sungjune Park, Se-Hoon Park

**Affiliations:** 1 ICT Device Packaging Research Center, Korea Electronics Technology Institute (KETI), Seongnam 13509, Republic of Korea; swook@keti.re.kr (S.K.); suin9804@keti.re.kr (S.C.); psb408@keti.re.kr (S.P.); hjnam1203@keti.re.kr (H.N.); 2 School of Chemical Engineering, Sungkyunkwan University, Suwon 16419, Republic of Korea; sungjunepark@skku.edu; 3 Department of Electronic Engineering, Hanyang University, Seoul 04763, Republic of Korea

**Keywords:** dry-film photoresists, pattern collapse, surface treatment, surface chemistry, mechanical properties, redistribution layer, interposers, semiconductor packaging

## Abstract

Dry-film photoresists (DFRs) are susceptible to pattern collapse arising from hydrophilic water–DFR interactions after the development process. Although wet surface treatment (rinsing) has been proposed to increase the hydrophobicity and prevent collapse, the associated surface chemical and mechanical changes remain insufficiently understood. In this study, the effects of these changes on pattern collapse were systematically analyzed using rinses containing tetramethylammonium chloride (TMAC), cetyltrimethylammonium chloride (CTAC), or BHA hydrochloride (BHAC). The surface free energy decreased after rinsing, confirming the increased hydrophobicity. During photolithography, pattern collapse was worsened by TMAC or CTAC but prevented by BHAC. The mechanical strength was degraded by TMAC or CTAC but enhanced by BHAC, as observed by nanoindentation. Diffusion of cations into the DFR was confirmed by confocal Raman microscopy, while changes in the chemical structure and apparent crosslink density arising from cation–DFR interactions were evaluated by Fourier transform infrared spectroscopy and swelling tests, respectively. In particular, BHAC treatment enhanced hydrogen-bonding in the DFR, which was associated with a higher apparent crosslink density and improved mechanical robustness. Finally, the BHAC-containing rinse was selected as the optimal rinse and used to fabricate a panel-level redistribution layer interposer. The interposer was successfully fabricated, exhibiting an insertion loss magnitude below 1.4 dB over 0–40 GHz. These results highlight that pattern collapse of the DFR can be effectively prevented using a BHAC-containing rinse during fine patterning.

## 1. Introduction

High-performance computing, automotive, and artificial intelligence applications all rely on die-to-die high-speed interfaces [1,2,3]. High-density I/O is implemented to realize such interfaces, and continuous efforts are being made to route I/O on a highly integrated redistribution layer (RDL) [4,5]. This trend in the semiconductor packaging industry is addressed in 2.xD packaging by scaling the pitch of the RDL [6,7]. The RDL interposer consists of thin polymer dielectrics and Cu traces; therefore, a reduction in the RDL feature size has a direct and significant impact on the performance of high-speed interfaces [8,9]. At the same time, interposer scaling is associated with tradeoffs in yield, area efficiency, and cost, making further miniaturization more difficult [10].

A wafer-level process (WLP) or panel-level process (PLP) is used to fabricate the RDL interposer. In WLP, the processable area is relatively small, and the wafer edge is rounded, which results in a lower yield in the dicing step. However, the damascene process, including chemical-mechanical planarization, can be employed, which is advantageous for implementing fine-pitch RDL [11]. In PLP, the processable area is larger and the panel has rectangular corners; therefore, the dicing yield is higher. However, the RDL is usually fabricated by a semi-additive process (SAP), which imposes a limitation on RDL scaling. Nevertheless, as the number of chiplets mounted on the interposer increases, an interposer with a larger area is required. For such large form factors, PLP is more suitable than WLP. This has created a continuous demand for PLP capable of realizing high-speed interfaces over large areas [4,12,13,14].

The difficulty of RDL scaling in PLP arises not only from the process architecture, but also the materials used. Photoresists, which are acrylate-based polymers, are essential for fabricating fine-pitch RDLs. Both liquid photoresists (LPRs) and dry-film photoresists (DFRs) can be used in the SAP. LPRs are applied to a substrate by spin coating, slit coating, and other related techniques; however, a uniform film thickness is difficult to obtain over a large panel, and the process is sensitive to particles [15,16]. In contrast, the DFR can be laminated, allowing the formation of a uniform film on the substrate. In addition, the PET film covering the resin makes the process less sensitive to contamination [17,18]. Moreover, because a DFR is typically thicker than an LPR, an RDL with a higher aspect ratio can be formed. In such an RDL, the cross-sectional area of the copper traces is large, resulting in a lower insertion loss of the circuit. Thus, DFR is more advantageous than LPR in terms of processability and electrical performance [19].

However, high-aspect-ratio DFR patterns are susceptible to collapse. Pattern collapse is a representative failure mode of the DFR; once it occurs, open- or short-circuit defects can be generated during the electroplating of the RDL. Consequently, pattern collapse has become a major challenge in RDL scaling [20,21,22]. Pattern collapse is driven by capillary forces arising from water–DFR interactions in high-aspect-ratio patterns. The water–DFR interaction is attributed to the carboxylate anions generated on the DFR surface during alkaline development. Pattern collapse occurs when the capillary force exceeds the mechanical strength of the DFR. Therefore, pattern collapse can be effectively prevented when the capillary force arising from the water–DFR interaction is sufficiently reduced below the mechanical strength of the DFR [23,24,25].

Namatsu et al. theoretically derived the condition for pattern collapse and calculated the critical tensile stress from the capillary force. According to previous studies, pattern collapse occurs when the capillary force exceeds the mechanical strength of the pattern [26]. Drechsler et al. reported that a wet surface treatment containing cetyltrimethylammonium chloride (CTAC) applied to the LPR after development converted the hydrophilic surface to a more hydrophobic surface and mitigated pattern collapse [27,28]. They investigated the mechanism of hydrophobic surface modification using surface-charge analysis; however, the effect of the rinse treatment on the mechanical strength was not discussed. Although LPR and DFR serve the same lithographic function, they differ in their material form, film formation process, thickness, and potentially in their polymer formulation and network structure. Therefore, the response of the resist network to an absorbed cationic species cannot necessarily be assumed to be identical between LPR and DFR. In the previous LPR studies, the beneficial effect of CTAC was mainly interpreted in terms of hydrophobic surface modification, whereas changes in the mechanical properties of the resist were not evaluated. In the present DFR system, CTAC also increased the surface hydrophobicity; however, nanoindentation showed a substantial decrease in the mechanical properties, and plastic deformation was observed in the patterned DFR. These results indicate that for the present DFR, the mechanical degradation induced by CTAC outweighed the beneficial reduction in the water–DFR interaction, resulting in more severe pattern collapse. Sharp et al. suggested a method to improve the robustness of LPR by introducing crosslinking agents into the resin. However, this approach has limited applicability to other photoresists, including DFR [29].

The current study aimed to systematically analyze the changes in the surface chemical and mechanical properties of DFR following treatment with different rinses and to investigate their effects on pattern collapse. Aqueous solutions containing the following cationic compounds were evaluated as rinses: tetramethylammonium chloride (TMAC), CTAC, and BHA hydrochloride (BHAC). Changes in the surface chemical properties of the DFR after rinsing were analyzed to assess the hydrophobic surface modification. The surface free energy (SFE) of the DFR was quantified via contact angle analysis using three standard liquids. The mechanical properties of the DFR were characterized by nanoindentation based on load–displacement curves obtained by pressing a diamond tip into the DFR [30]. The relationships between the hydrophobicity, mechanical properties, and pattern collapse were investigated. The diffusion of cations into the DFR was identified using confocal Raman microscopy. Cation-induced changes in the polymer network, including changes in the chemical structure and crosslink density, were analyzed using Fourier transform infrared (FTIR) spectroscopy and swelling tests, respectively. A relationship between the changes in the polymer network and variations in the mechanical properties was demonstrated. Based on these results, the BHAC-containing rinse was determined to be optimal and applied to fabricate a panel-level RDL interposer. Additionally, the electrical properties of the interposer were evaluated and compared with those obtained by electromagnetic simulations.

## 2. Materials and Methods

### 2.1. Materials

Rinses A, B, and C were prepared by dissolving TMAC (Sigma-Aldrich, St. Louis, MO, USA), CTAC (Sigma-Aldrich, St. Louis, MO, USA), and BHAC, respectively, in deionized water (DIW). BHAC is a hydrochloride salt of an aryl-containing bis(hydroxyethyl)amine derivative. As illustrated in Table 1, R denotes the C6–C10 aryl group with a urethane linkage.

Sealed glass baths used to contain the rinses were first cleaned with isopropyl alcohol (IPA; Sigma-Aldrich, St. Louis, MO, USA) and DIW for 1 min each. As described in Table 2, rinses A–C were formulated at a concentration of 10 ± 0.5 mM. To prepare the rinses, 500 mL of DIW was mixed with the corresponding cation, and the solution was stirred at 300 rpm for 5 min. An additional 500 mL of DIW was added, and the mixture was stirred again at 300 rpm for 5 min, yielding a total volume of 1000 mL. The pH values of the rinses were confirmed to be 7.0 ± 0.5.

### 2.2. Preparation of Samples

A 7-μm-thick negative-tone DFR (RY-5107UT; Resonac, Tokyo, Japan) was laminated onto a 4-inch-diameter Si wafer at 120 °C, and the DFR was exposed to UV light using a mask aligner (MDA-400S; MIDAS System, Daejeon, Republic of Korea) with a wavelength of 365 nm (i-line) at an exposure dose of 200 mJ/cm^2^. The exposed DFR was then developed in a 1 wt% Na_2_CO_3_ aqueous solution for 60 s, treated in the prepared rinses for a fixed time, and finally cleaned in DIW for 10 s. The rinse treatment time was controlled within ±1 s. In addition, the interval between rinse treatment and subsequent deionized water cleaning was controlled to within 2 s.

### 2.3. Characterization

The SFE of the DFR was evaluated to quantify the extent of hydrophobic surface modification and determine the optimal rinse treatment time. The static contact angles (SCAs) of DIW, ethylene glycol, and n-hexane on the DFR surface were measured by the sessile drop method using a contact-angle analyzer (Phoenix 300; SEO, Suwon, Republic of Korea). Based on the measured SCAs, SFE and its dispersive and polar components were calculated using the Owens–Wendt–Rabel–Kaelble (OWRK) method. The optimal rinse treatment time was defined as the time at which SFE reached a plateau. All subsequent analyses were performed using the samples treated with each rinse for the optimal treatment time.

The applicability of the rinses for the fine patterning of DFR was evaluated using photolithography. Patterns with critical dimensions of 2, 3, and 4 µm were formed using a mask aligner (MIDAS MDA-400S). The extent of pattern collapse in the DFR was determined using scanning electron microscopy (SEM; GENESIS-1000; EMCRAFTS, Hanam, Republic of Korea) after each rinse treatment.

The mechanical properties of the DFR were characterized using nanoindentation after rinsing. The force–depth curves of the DFR were obtained using a nanoindenter (HM2000; Bruker, Billerica, MA, USA). The elastic modulus, indentation hardness, and resilience were calculated from these curves.

Confocal Raman microscopy (Alpha300 Apyron; WITec, Ulm, Germany) was used to measure the Raman spectra of the DFR as a function of depth after rinsing. C–N single-bond vibrations were observed at Raman shifts of 750 and 960 cm^−1^, and the diffusion profiles of the ammonium cations were obtained based on the peak area at 750 cm^−1^. Furthermore, the diffusivities of the cations were calculated by nonlinear fitting of the diffusion profiles based on Fick’s second law of diffusion.

FTIR spectroscopy was used to investigate the chemical bonds between the cations and DFR after rinsing. Spectra were acquired using an FTIR microscope (LUMOS II; Bruker, Billerica, MA, USA). The spectra were then compared across the samples to identify changes in the peak positions and areas, and the corresponding chemical bonds were assigned based on the observed peaks.

The swelling ratio and crosslink density of the DFR were determined by swelling tests following the rinse treatment. First, the density of the DFR was measured. Subsequently, the DFR was immersed in IPA (Sigma-Aldrich, St. Louis, MO, USA) for 1 h, and the weight of the swollen DFR was measured after excess IPA was removed from its surface. The DFR was then dried in an oven at 110 °C for 1 h, and its dry weight was measured. The swelling ratio was calculated using the measured weights and DFR density. The crosslink density was subsequently calculated from the swelling ratio using the Flory–Rehner equation:

### 2.4. Application of Optimal Rinse

Based on the above experiments, the optimal rinse was selected and used to fabricate a panel-level RDL interposer test vehicle using the SAP. Using a photoimageable dielectric (PID; WPR-1201; JSR, Tokyo, Japan), DFR (Resonac RY-5107UT), and the optimal rinse, RDLs with line/space (L/S) dimensions ranging from 2 µm/2 µm to 4 µm/4 µm were formed on a 100 mm × 100 mm copper-clad laminate (CCL) panel. Subsequently, the interposer was inspected using an optical microscope (MX-61; Olympus, Tokyo, Japan) and laser scanning microscope (VK-X3000; KEYENCE, Osaka, Japan).

Electrical tests of the interposer were performed, and the test results were compared with the trends of 3D electromagnetic (EM) simulations. The insertion loss (signal loss of the circuit) as a function of the RDL L/S was measured using a vector network analyzer over the frequency range of 100 kHz to 40 GHz. A GSGSG-type (ground-signal-ground-signal-ground) microprobe with a 50 µm pitch (Picoprobe; GGB Industries, Naples, FL, USA) was used for all the measurements to ensure consistent probing conditions.

## 3. Results and Discussion

### 3.1. Surface Chemical Properties of DFR

The SCA and SFE of the DFR after rinse treatment were analyzed. Based on the OWRK model [31], the SFE of the DFR can be expressed as Equation (1):

(1) $$\gamma_{S}\;=\;\gamma_{S}^{d}\;+\;\gamma_{S}^{p}$$

where γ is the SFE, the subscript S denotes the solid (DFR), and the superscripts d and p represent the dispersive and polar components of the SFE, respectively. In addition, the SCAs of each standard liquid were related to the dispersive and polar components of the SFE. The dispersive and polar components were then calculated from Equations (2) and (3) using linear regression:

(2) $$\gamma_{\mathrm{Li}}\;\left(1\;+\;\cos\theta_{\mathrm{Li}}\right)\;=\;2\left(\sqrt{\gamma_{S}^{d}\;\gamma_{\mathrm{Li}}^{d}}\;+\;\sqrt{\gamma_{S}^{p}\;\gamma_{\mathrm{Li}}^{p}}\right)$$

(3) $$\frac{\gamma_{\mathrm{Li}}\;\left(1+\cos\theta_{\mathrm{Li}}\right)}{2\sqrt{\gamma_{\mathrm{Li}}^{d}}}=\sqrt{\gamma_{S}^{d}}+\sqrt{\gamma_{S}^{p}}\frac{\sqrt{\gamma_{\mathrm{Li}}^{p}}}{\sqrt{\gamma_{\mathrm{Li}}^{d}}}\Rightarrow \;Y=\sqrt{\gamma_{S}^{d}}+\sqrt{\gamma_{S}^{p}}\;X$$

where the subscript Li is the i-th standard liquid, and θ represents the measured SCA of the standard liquid on the DFR. The surface chemical properties of the standard liquids are listed in Table 3 [32,33].

**Figure 1:**
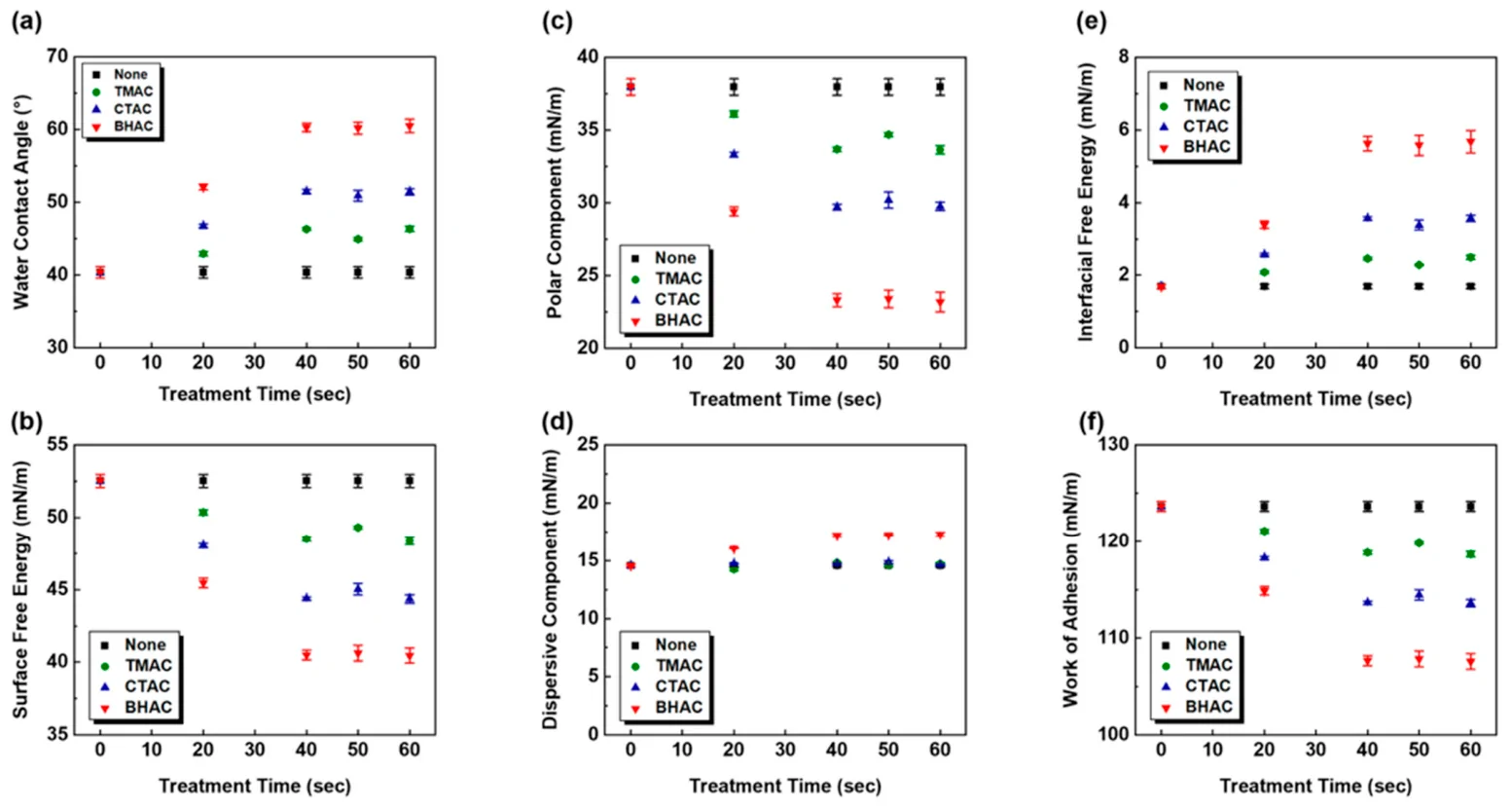
Surface chemical properties of DFR after rinse treatment: (**a**) water contact angle, (**b**) surface free energy, (**c**) polar component, (**d**) dispersive component, (**e**) interfacial free energy, and (**f**) work of adhesion.

As shown in Table 4 and Figure 1a,b, the water contact angle increased and the SFE decreased with increasing treatment time compared with no treatment, indicating that the DFR surface became more hydrophobic after rinsing. The largest increase in the water contact angle and largest decrease in the SFE were observed after BHAC treatment, followed by CTAC and TMAC, confirming that BHAC produced the strongest hydrophobic surface modification.

As shown in Figure 1c, the polar component of the SFE decreased markedly with increasing treatment time. This decrease is presumed to result from the neutralization of carboxylate anions on the DFR surface after development. The magnitude of the decrease in the polar component followed the order: BHAC > CTAC > TMAC, which was presumed to be associated with the shielding of the polar groups on the DFR surface by the hydrophobic groups of CTAC and BHAC. BHAC was presumed to provide a stronger shielding effect on the polar groups than CTAC, as supported by the increase in the dispersive components shown in Figure 1d [28,34,35].

The interfacial free energy (IFE) at the water–DFR interface was calculated using Equation (4):

(4) $$\gamma_{\mathrm{SL}1}\;=\;\gamma_{S}-\gamma_{L1}\cos\theta$$

where γ_SL1_ denotes the IFE at the water–DFR interface, γ_s_ is the SFE of DFR, γ_L1_ is the SFE of DIW, and θ represents the contact angle of DIW, respectively. As illustrated in Figure 1e, the IFE was the highest after BHAC treatment, followed by CTAC and TMAC. For water, a higher IFE indicates that the formation of the water–DFR interface is less favorable, corresponding to lower wettability and a more hydrophobic surface [36].

Furthermore, the work of adhesion of the water–DFR interface was obtained using Equation (5).

(5) $$W_{A}=\gamma_{S}+\gamma_{L1}-\gamma_{\mathrm{SL}1}$$

where W_A_ is the work of adhesion. W_A_ followed the order TMAC > CTAC > BHAC, which was opposite to the trend of IFE and indicated lower interfacial stability after BHAC treatment. Thus, the lowest work of adhesion was obtained after BHAC treatment.

Based on these results, rinsing for 40 s, when the water contact angle and SFE plateaued, was determined to be optimal. For BHAC treatment, the water contact angle and SFE remained nearly unchanged from 40 to 60 s, indicating that the surface modification was stable within this treatment-time range. Therefore, 40–60 s can be regarded as a stable treatment-time window for hydrophobic surface modification under the conditions investigated in this study. However, subsequent patterning, mechanical, and structural analyses were performed at the selected treatment time of 40 s; therefore, the overall process window for fine patterning cannot be quantitatively defined from the present data. In addition, the rinse concentration was fixed at 10 mM throughout this study to isolate the influence of cation chemistry. Accordingly, a quantitative concentration process window could not be established in the present study and should be evaluated in future work.

### 3.2. Photolithography Tests of DFR

**Figure 2:**
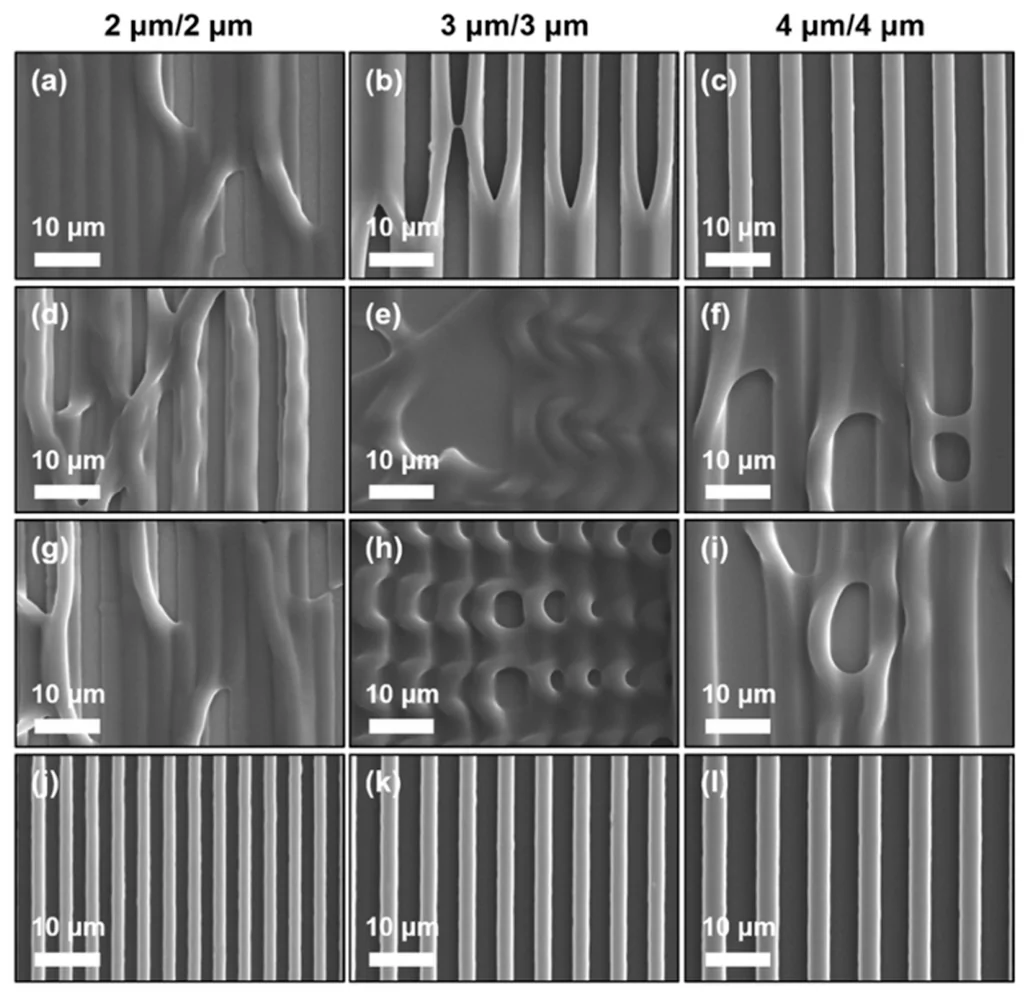
Representative SEM images of DFR patterns after rinse treatment: (**a**–**c**) no treatment, (**d**–**f**) TMAC, (**g**–**i**) CTAC, and (**j**–**l**) BHAC for L/S = 2 μm/2 μm to 4 μm/4 μm.

Rinses A–C were applied to the DFR during the photolithography process, and the resulting patterns were observed using SEM to evaluate pattern collapse. As shown in Figure 2, pattern collapse was observed for the untreated DFR at L/S = 2 μm/2 μm to 3 μm/3 μm, whereas the DFR treated with TMAC or CTAC exhibited pattern collapse across all evaluated patterns. In contrast, no pattern collapse was observed for DFR treated with BHAC under any of the evaluated patterns.

The more severe pattern collapse observed after treatment with TMAC or CTAC was contrary to the general expectation that pattern collapse could be prevented by suppressing the water–DFR hydrophilic interaction. However, plastic deformation such as stretching or tearing of the DFR was observed in the patterns treated with TMAC and CTAC. Therefore, not only the surface chemical properties but also the mechanical strength of the DFR was presumed to change after the rinse treatment.

### 3.3. Mechanical Properties of DFR

Nanoindentation was performed to investigate the change in the mechanical strength of the DFR after rinsing, where each rinse was applied for 40 s. Indentation force–depth curves were obtained, and the elastic modulus and indentation hardness were calculated from the curves. The elastic modulus is a mechanical property that remains relatively constant with the indentation depth and represents the bulk strength of a material. In contrast, indentation hardness is a depth-dependent mechanical property that reflects the surface strength of a material, particularly at shallow indentation depths. The resilience of the DFR was calculated from the measured elastic modulus and indentation hardness using Equations (6) and (7):

(6) $$\sigma_{y}\;=\;\frac{H_{\mathrm{IT}}}{3}$$

(7) $$\mathrm{Resilience}=\frac{\sigma_{y}^{2}}{2E}=\frac{H_{\mathrm{IT}}^{2}}{18E}$$

where σ_y_ is the yield stress, H_IT_ is the indentation hardness, and E is the elastic modulus. Equation (6) represents the relationship between the yield strength and indentation hardness, as proposed by Tabor. In addition, resilience can be determined from the indentation hardness and modulus because it can be described using the yield strength, as expressed by Equation (7). Resilience is defined as the energy per unit volume that can be absorbed before permanent deformation (i.e., plastic deformation). Therefore, resilience can be considered as an index of the elastic limit of the DFR [37,38].

**Figure 3:**
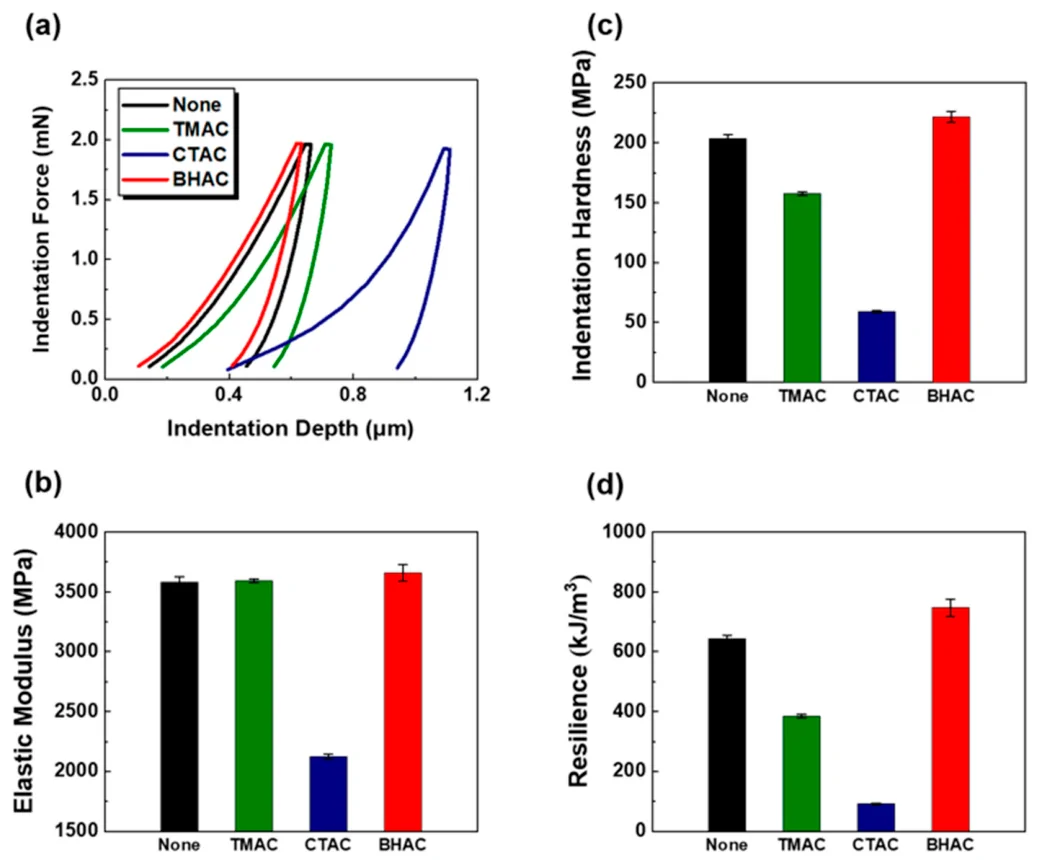
Nanoindentation test results: (**a**) indentation force–depth curve, (**b**) elastic modulus, (**c**) indentation hardness, and (**d**) resilience.

As shown in Figure 3a, when the same indentation load was applied, the indentation depth was the lowest for BHAC, followed by no treatment, TMAC, and CTAC.

As shown in Figure 3b–d, the elastic modulus increased slightly after BHAC treatment, whereas a significant decrease was observed after CTAC treatment. The indentation hardness decreased in the order CTAC > TMAC, whereas a noticeable increase was observed after BHAC treatment. The same trend was observed for resilience, with a larger decrease after CTAC treatment than after TMAC treatment and a noticeable increase after BHAC treatment.

When the resilience is sufficiently reduced, the capillary force generated by the surface tension of water exceeds the elastic limit, resulting in an irreversible pattern collapse. The significant decrease in the resilience after TMAC or CTAC treatment was consistent with the plastic deformation observed in the patterns. Therefore, although the DFR treated with TMAC or CTAC became more hydrophobic, its mechanical strength degraded, making it more susceptible to pattern collapse. In contrast, DFR treated with BHAC became more hydrophobic and its mechanical strength was enhanced, making it more resistant to pattern collapse [39].

Namatsu et al. theoretically showed that pattern distortion occurs when the spacing between adjacent patterns becomes smaller than a critical value proportional to the square of the pattern aspect ratio, expressed as $D<k{\left(H/W\right)}^{2}$, where D is the spacing between adjacent patterns, H is the pattern height, W is the pattern width, and k is a constant dependent on the dynamic contact angle [26]. This relationship indicates that pattern collapse is strongly influenced by both pattern geometry and the capillary interaction generated during the drying process.

However, an absolute critical collapse dimension was not calculated in the present study because the current experimental quantities are insufficient to determine the corresponding critical parameter for the present DFR system. The surface free energy measured in this study characterizes the solid DFR surface, whereas the nanoindentation-derived resilience represents the elastic energy density that can be absorbed before permanent deformation. Therefore, these quantities cannot be directly substituted into the reported criterion to determine the critical collapse dimension without establishing an appropriate relationship between the measured surface and mechanical properties and the model parameters. In addition, the static contact angle measured on a flat DFR surface may not fully represent the local wetting and meniscus conditions of the patterned sidewalls during drying. Therefore, direct calculation using the present dataset could lead to a quantitatively misleading prediction. Nevertheless, the experimental results clearly demonstrate that hydrophobic surface modification alone cannot explain the observed pattern collapse behavior because TMAC and CTAC reduced the water–DFR interaction while simultaneously degrading the mechanical properties of the DFR.

### 3.4. Diffusion of Cations in DFR

**Figure 4:**
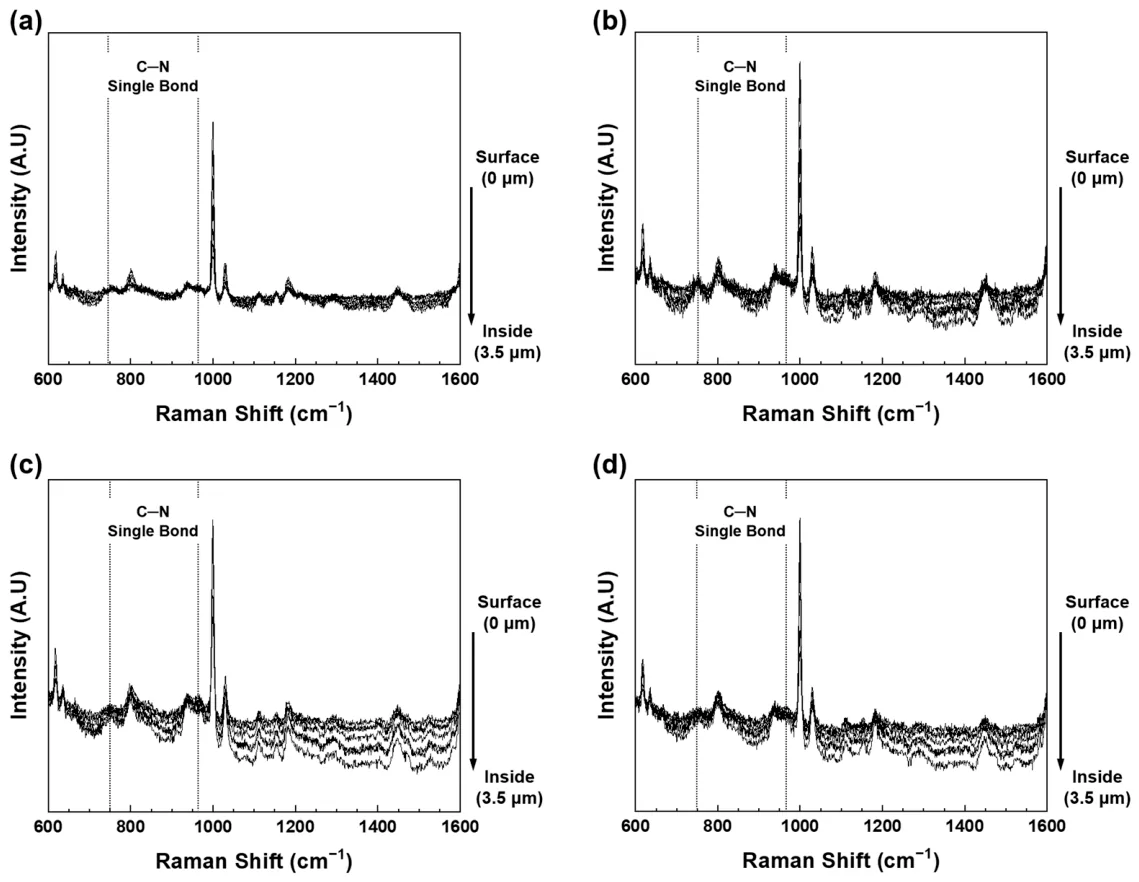
Raman spectra of DFR after rinse treatment: (**a**) no treatment, (**b**) TMAC, (**c**) CTAC, and (**d**) BHAC.

The changes in the mechanical strength of the DFR after rinsing indicated that the bulk properties of the DFR were altered. Therefore, the cations were presumed to diffuse into the DFR during the rinse treatment. Confocal Raman microscopy was used to verify cation diffusion into the DFR. As illustrated in Figure 4, peaks assigned to C–N single-bond vibrations associated with the cations were observed at Raman shifts of 750 and 960 cm^−1^. For quantitative analysis, the peak area at 750 cm^−1^ was integrated as a function of depth. Because the peak at 960 cm^−1^ overlapped with a peak from the Si wafer, it was excluded from the integration. The diffusivity of the cations was obtained by nonlinear fitting of the normalized concentration profile (calculated from the peak areas) to the solution to Fick’s second law. The normalized cation concentration was calculated using Equation (8):

(8) $$\mathrm{Normalized}\;\mathrm{Conc}.\;=\;\frac{A(x)-A_{\mathrm{None}}\left(x\right)}{A(0)-A_{\mathrm{None}}\left(0\right)}\;=\;\mathrm{erfc}\left(\frac{x}{2\sqrt{\mathrm{Dt}}}\right)$$

where x denotes the scanning depth; A(x) and A_None_(x) denote the areas of the rinse-treated and untreated DFR at depth x, respectively; A(0) and A_None_(0) denote the areas of the rinse-treated and untreated DFR at the surface, respectively; erfc denotes the complementary error function, which represents the solution to Fick’s second law; D denotes the diffusivity; and t denotes the optimal rinse treatment time [40,41,42].

**Figure 5:**
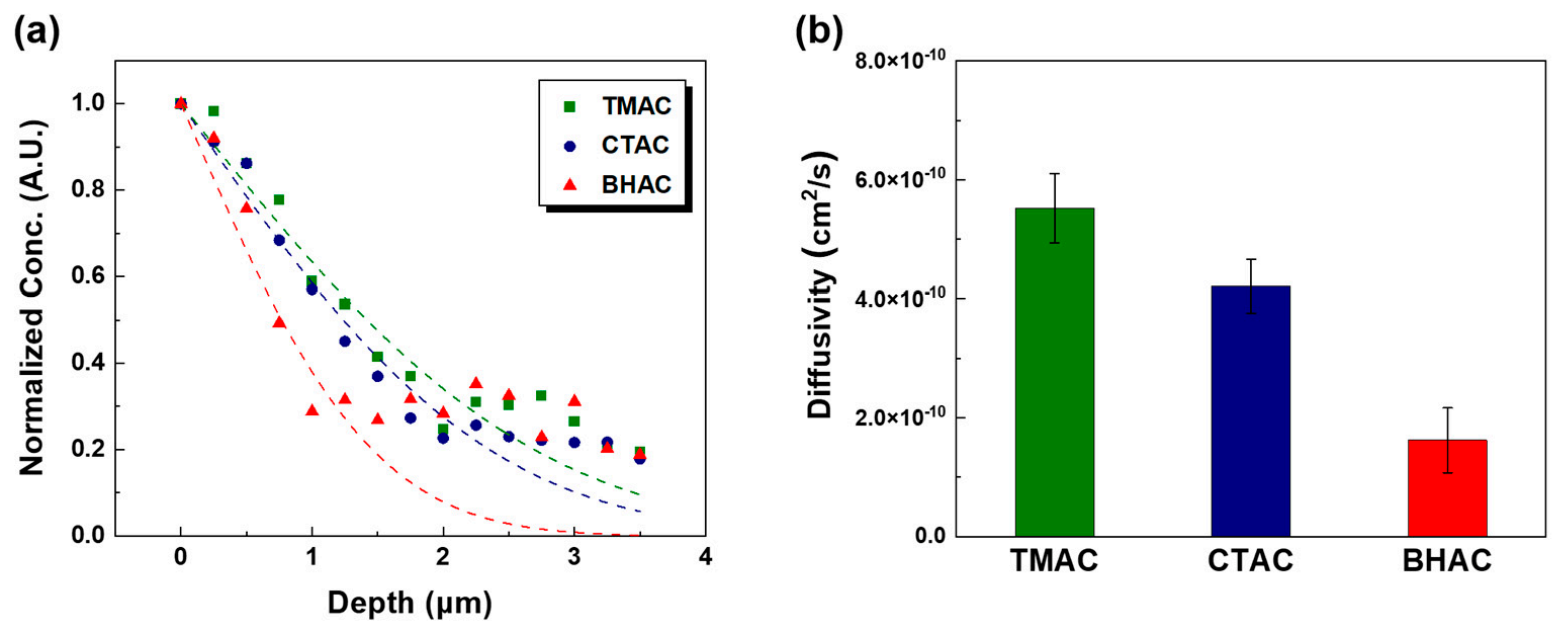
Integration results of the peaks at 750 cm^−1^: (**a**) normalized concentration–depth profile and (**b**) diffusivity of cations.

As shown in Figure 5, cations were observed to a depth of 3.5 µm in the DFR after rinse treatment. The cation concentration decreased with increasing depth, which is consistent with the concentration profile described by the complementary error function. The diffusivities obtained by nonlinear fitting decreased in the following order: TMAC > CTAC > BHAC. Among the three cations, the highest diffusivity of TMAC was attributed to its smaller molecular size. The lower diffusivity of CTAC compared with that of TMAC was attributed to its larger molecular size owing to its hydrophobic moiety. BHAC exhibited the lowest diffusivity, which is attributed to hydrogen bonding between the hydroxyl groups of its diol groups and the carbonyl groups in the DFR, increasing the resistance to diffusion within the DFR.

Meanwhile, although CTAC exhibited a lower diffusivity than TMAC, it caused more pronounced degradation of the mechanical properties. This result indicates that the magnitude of the plasticizing effect is not necessarily positively correlated with cation diffusivity. Diffusivity primarily describes the rate of cation penetration into the DFR, whereas the resulting changes in mechanical properties are also influenced by the molecular structure of the cation and the strength of cation–polymer interactions. Therefore, the changes in the mechanical robustness of the DFR should be interpreted by considering both cation diffusivity and cation–polymer interactions [43,44].

### 3.5. Analysis of Chemical Bonding

The differences in mechanical strength after rinsing were considered to originate from changes in the polymer network induced by diffused cations. Therefore, the FTIR spectra of DFR after rinse treatment were investigated.

**Figure 6:**
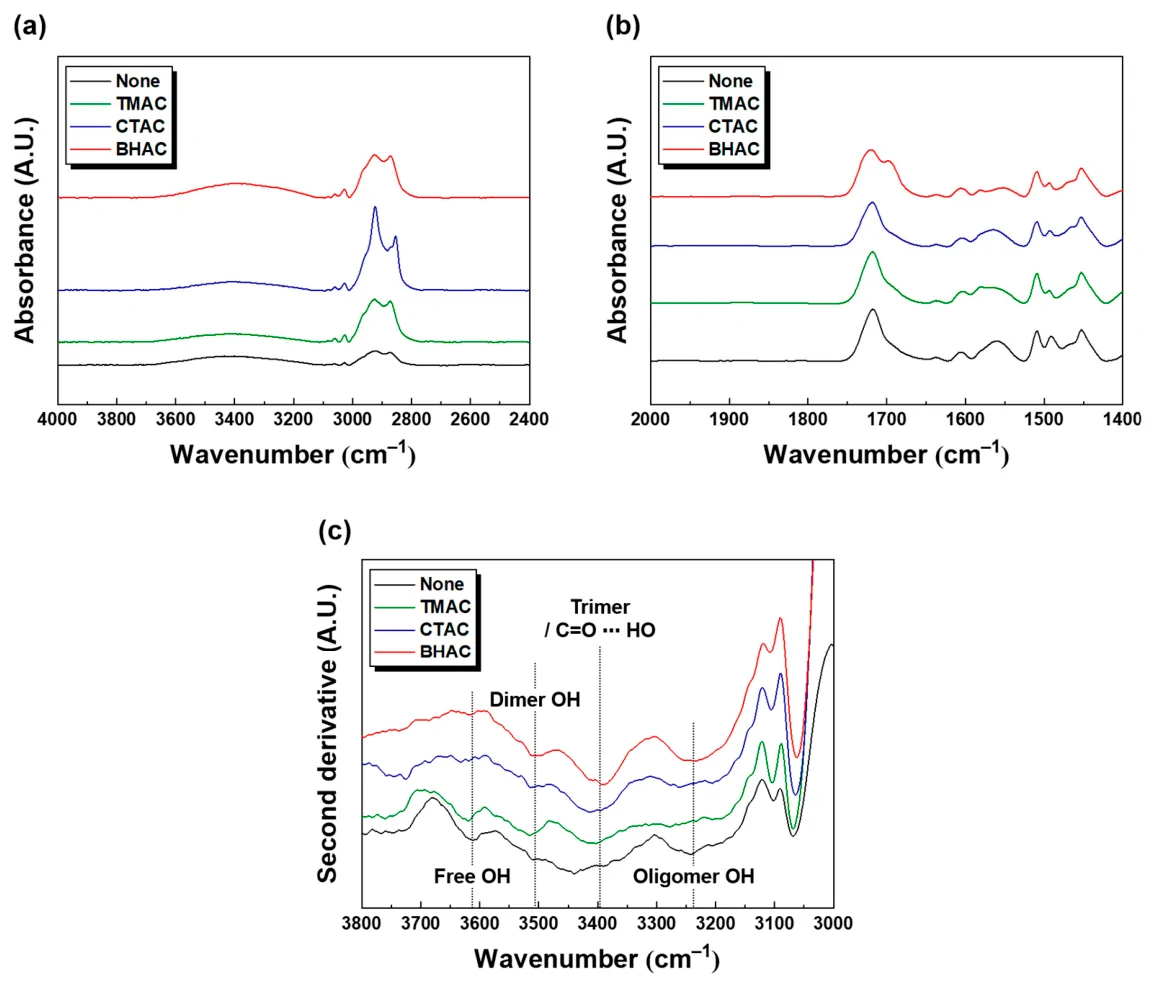
FTIR spectra of the DFR after rinse treatment: (**a**) wavenumber range of 4000–2400 cm^−1^, (**b**) wavenumber range of 2000–1400 cm^−1^, and (**c**) second derivative spectra of OH stretching band.

As shown in Figure 6a, the CH stretching peaks at 2800–3050 cm^−1^ increased after treatment with TMAC, CTAC, or BHAC, indicating an increase in hydrophobic moieties in the DFR. A significant increase in the OH stretching band at 3700–3100 cm^−1^ was observed only after BHAC treatment. This broad band was assigned to hydrogen-bonded OH groups. Specifically, the broad band contains overlapping contributions from OH···OH hydrogen bonds and C=O···HO hydrogen bonds [45,46,47]. In addition, as shown in Figure 6b, the C=O···HO peak at 1700 cm^−1^ increased only after BHAC treatment. These results suggest that C=O···HO hydrogen bonding may play a key role in the enhancement of mechanical strength induced by BHAC. Therefore, the broad band at 3100–3700 cm^−1^ was deconvoluted to investigate the individual contribution of each type of hydrogen bond [48].

According to Mitani et al. and Sato et al., the OH stretching band at 3100–3700 cm^−1^ can be deconvoluted, from higher to lower wavenumbers, into a free OH peak that does not participate in hydrogen bonding; peaks associated with hydrogen-bonded OH clusters, including dimers, trimers, and oligomers; and a peak corresponding to C=O···HO. Specifically, Mitani et al. assigned the peaks at 3620, 3497, 3378, and 3237 cm^−1^ to the free OH, OH dimer, overlapping OH trimer and C=O···HO hydrogen bonds, and OH oligomer, respectively. Similarly, as illustrated in Figure 6c, four local minima were apparent at nearly identical wavenumbers in the second-derivative spectra of the OH stretching band, indicating that the band can be resolved into four components. Based on the peak assignment reported by Mitani et al. and the second-derivative spectra, the wavenumbers at 3610, 3506, 3391, and 3240 cm^−1^ were assigned to free OH, dimeric OH, overlapping trimeric OH and C=O···HO hydrogen bonds, and oligomeric OH, respectively [49,50,51].

**Figure 7:**
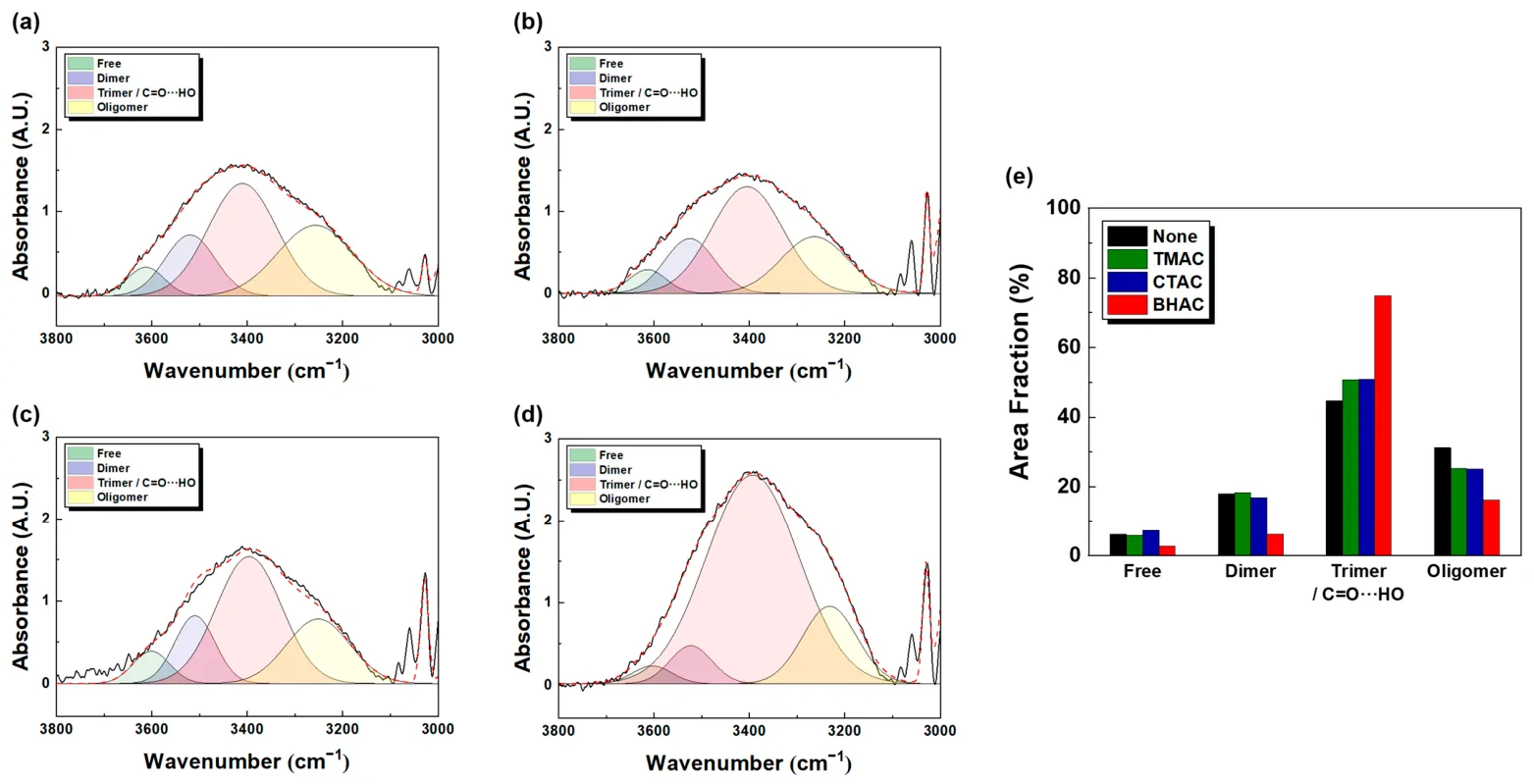
Deconvolution of the OH stretching band: (**a**) no treatment, (**b**) TMAC, (**c**) CTAC, (**d**) BHAC, and (**e**) area fraction of OH peaks.

As shown in Figure 7, the OH stretching band was deconvoluted into four hydrogen bonding peaks using Gaussian fitting. No significant changes in the deconvoluted peak areas were observed between the untreated DFR and DFR treated with TMAC or CTAC. In contrast, BHAC treatment led to noticeable decreases in the areas of the free OH and OH dimer, whereas the area of the overlapping peak assigned to the OH trimer and C=O···HO hydrogen bonds increased significantly.

**Figure 8:**
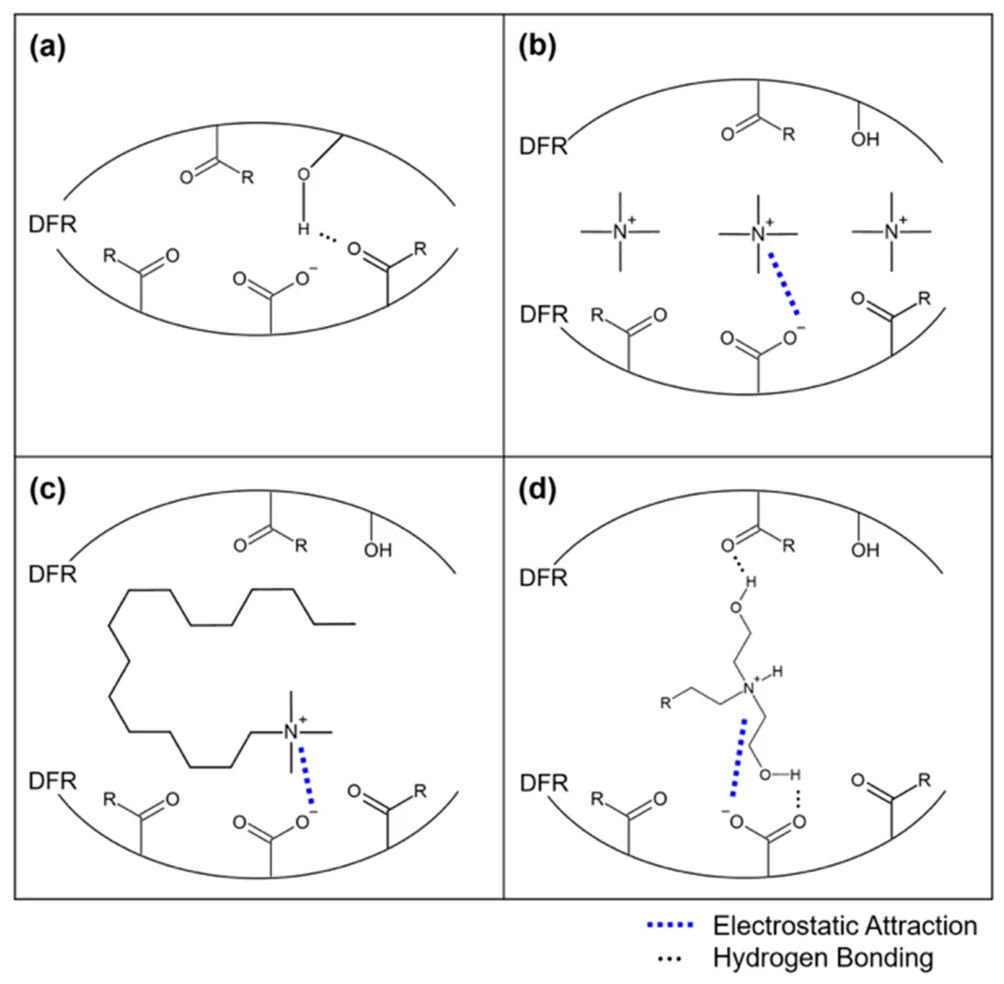
Schematic of the proposed polymer network after rinse treatment: (**a**) no treatment, (**b**) TMAC, (**c**) CTAC, and (**d**) BHAC.

These results are attributed to changes in the polymer network after rinsing. As shown in Figure 8, hydrogen bonding between the hydroxyl and carbonyl groups in the untreated DFR is presumed to contribute to its robust mechanical properties.

In contrast, TMAC and CTAC diffused into the DFR and were presumed to act as plasticizers, increasing the free volume and loosening the polymer network. In this loosened network, the formation of hydrogen bonds between the hydroxyl and carbonyl groups became less favorable, which was presumed to decrease the crosslink density. In particular, CTAC is expected to act as a stronger plasticizer than TMAC because its longer alkyl chain is considered to substantially increase the intermolecular distance in the DFR. As a result, CTAC was presumed to cause a greater reduction in the number of hydrogen bonds than TMAC [52,53,54].

Meanwhile, after BHAC treatment, the fraction of free OH decreased, whereas the fraction of OH trimers and C=O···HO hydrogen bonds increased significantly. These results indicate that more OH groups were involved in hydrogen bonding than before BHAC treatment, suggesting enhanced hydrogen-bonding interactions between the diol groups of BHAC and the carbonyl groups of DFR after BHAC treatment. Such enhanced intermolecular interactions may contribute to the mechanical strengthening observed after BHAC treatment [55,56].

### 3.6. Analysis of Crosslink Density

To investigate the changes in the crosslink density, swelling tests were performed after rinsing. The swelling ratio (Q), which represents volumetric expansion after solvent (IPA) absorption, was calculated using Equation (9):

(9) Q = wpρp+wSρSwpρp=1φ

where w_p_ and ρ_p_ denote the weight and density of the dried DFR, respectively; w_s_ and ρ_s_ denote the weight and density of IPA, respectively; and φ represents the volume fraction of DFR after solvent absorption [57]. φ could be theoretically related to the crosslink density of the DFR. According to the Flory–Rehner equation, the crosslink density (υ) was calculated from Equation (10):

(10) $$\upsilon \;=\;-\frac{\ln\left(1-\varphi \right)\;+\;\varphi \;+\;\chi \varphi^{2}}{V_{s}\left(\varphi^{1/3}-\frac{\varphi }{2}\right)}$$

where V_s_ denotes the molar volume of the IPA and χ is the Flory–Huggins interaction parameter, which describes the polymer–solvent affinity. Equation (10) yields an apparent crosslink density, including intermolecular interactions such as hydrogen bonding as well as covalent bonding [58,59,60]. χ was calculated from Equations (11) and (12):

(11) $$\chi \;=\;\frac{V_{s}{\left(\delta_{p}-\delta_{s}\right)}^{2}}{\mathrm{RT}}$$

(12) $$\delta_{p}=K\sqrt{\gamma }$$

where δ_p_ and δ_s_ are the solubility parameters of the DFR and IPA, respectively; γ is the surface free energy of the DFR after rinse treatment; and K is a proportionality constant (116 × 10^3^ m^−0.5^). The parameters described in Equations (9)–(12) are summarized in Table 5 [61,62,63].

**Figure 9:**
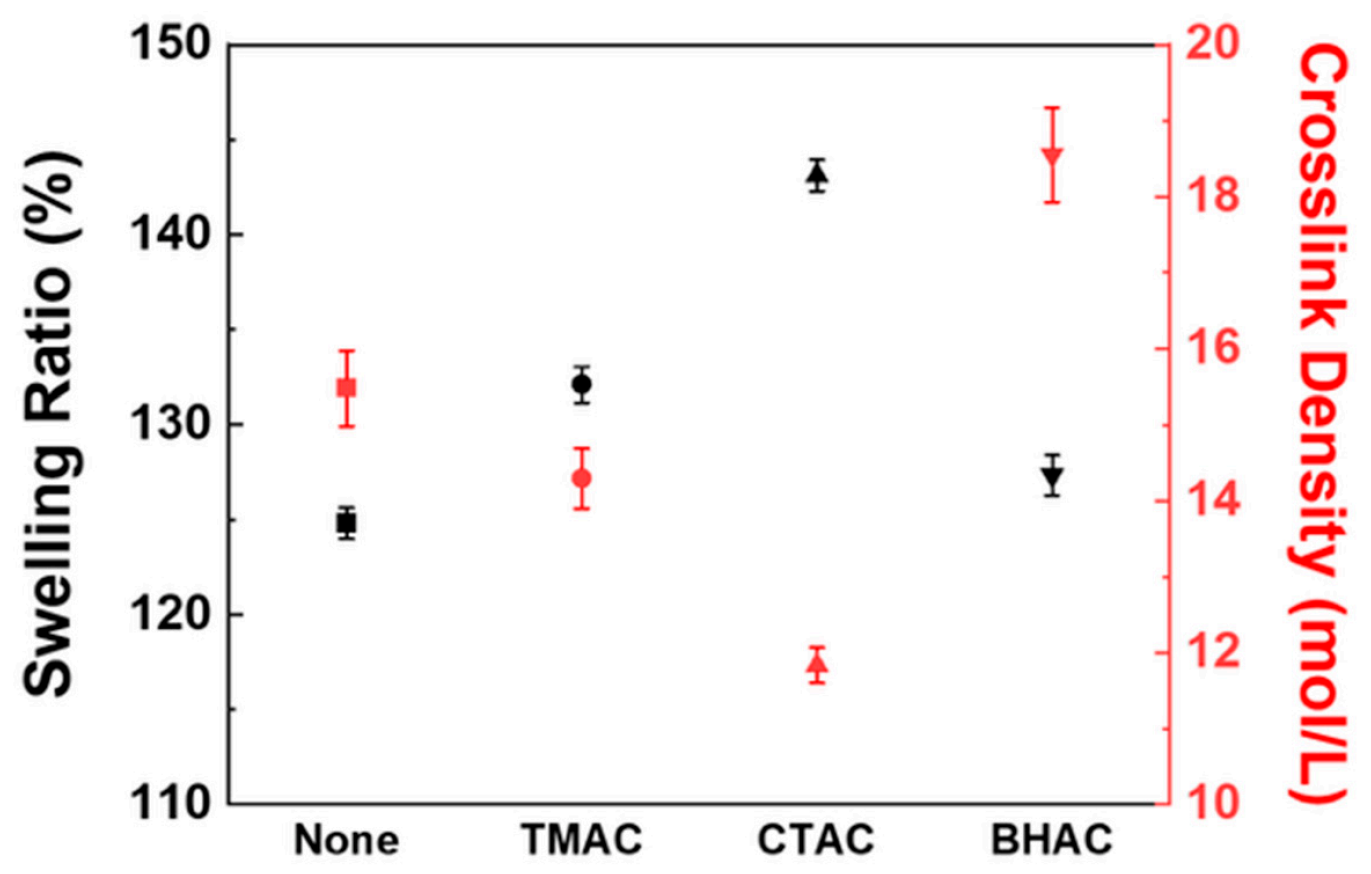
Swelling ratio and crosslink density of the DFR after rinse treatment.

As shown in Figure 9, the swelling ratio followed the order CTAC > TMAC > BHAC > untreated DFR, whereas the crosslink density followed the order BHAC > untreated DFR > TMAC > CTAC. The decrease in crosslink density after treatment with TMAC or CTAC is consistent with the interpretation that TMAC and CTAC act as plasticizers, suppressing intermolecular attraction within the DFR network. A greater decrease in crosslink density was observed after CTAC treatment than after TMAC treatment. This behavior is attributed to the longer alkyl chain of CTAC relative to the methyl groups of TMAC, as discussed above [54].

In contrast, BHAC treatment resulted in a higher apparent crosslink density than that of the untreated DFR. This trend was consistent with the enhanced hydrogen-bonding observed by the deconvoluted FTIR spectra. However, because the swelling test was conducted in IPA, a protic solvent, the persistence of specific BHAC–DFR hydrogen bonding during swelling cannot be directly established from the present data. Therefore, the results from the Flory–Rehner equation should be interpreted as indicating a higher apparent network constraint in the BHAC-treated DFR under the swelling conditions, rather than as direct evidence that specific BHAC–DFR hydrogen bonding remained intact during IPA immersion. The higher apparent network constraint was consistent with the enhanced mechanical robustness observed after BHAC treatment [55].

Based on these results, the changes in the mechanical strength of the DFR after the rinse treatment were found to be consistent with the changes in the polymer network. These findings underline that the mechanical strengthening of the DFR induced by cation diffusion is essential for preventing pattern collapse.

### 3.7. Application of Rinse C in Fabrication of Panel-Level RDL Interposer

**Figure 10:**
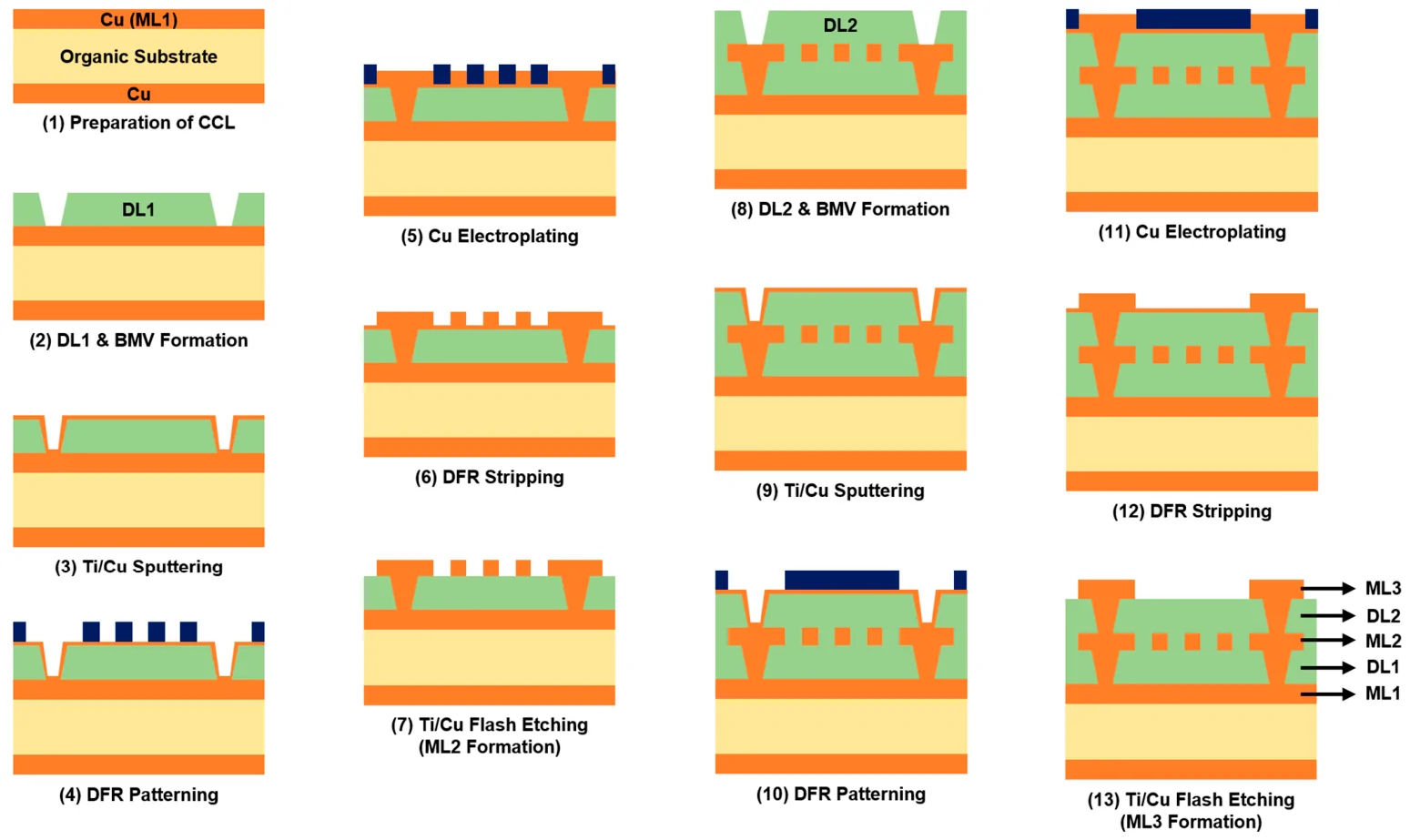
Fabrication process of the panel-level RDL interposer.

Based on the above comparison, rinse C was selected as the optimal solution and applied to the fabrication of a panel-level RDL interposer. The RDL was formed by a SAP on the 100 mm × 100 mm CCL panel, and the 7-μm-thick DFR was used in fine patterning. As shown in Table 6 and Figure 10, the interposer was designed and fabricated using two dielectric layers (DLs) and three metal layers (MLs).

In the fabrication of ML2, a Ti/Cu seed layer with a Ti/Cu thickness of 40/80 nm was deposited by sputtering. Subsequently, Cu traces with L/S ranging from 2 μm/2 μm to 4 μm/4 μm were formed on the DL1 by photolithography, Cu electroplating, and flash etching of the seed layer. The Cu thicknesses of ML1, ML2, and ML3 were 20 μm, 2 μm, and 3 μm, respectively. To implement blind micro via (BMV) with a diameter of 10 µm, a PID was employed as a dielectric material in the DLs. The PID was coated on the panel by automatic applicator with a thickness of 10 μm. During BMV formation using PID, an O_2_ plasma cleaning was also employed (300 W, 0.150 Torr, 50 sccm).

**Figure 11:**
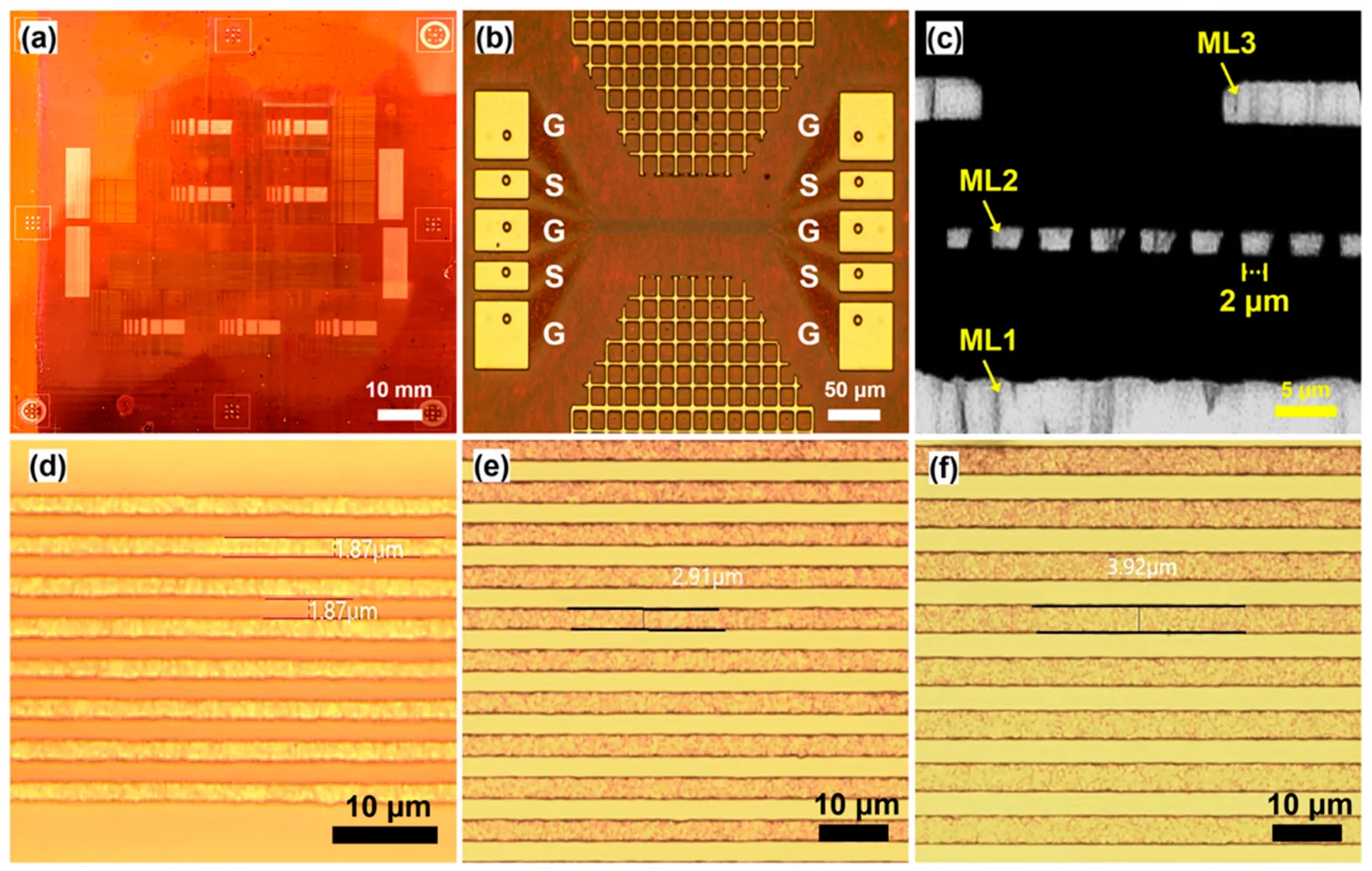
Representative images of panel-level RDL interposer: (**a**) 100 mm × 100 mm interposer, (**b**) representative optical microscopy image of electrical test pattern with GSGSG pad structures, (**c**) cross-sectional laser scanning microscopy image of 2 μm/2 μm RDL, and (**d**–**f**) optical microscopy images of RDL for L/S = 2 μm/2 μm to 4 μm/4 μm.

Figure 11 shows the fabricated interposer, which was successfully implemented as a three-layer RDL interposer. In addition, the RDL included Cu traces with GSGSG pad structures for electrical tests. The insertion loss (S21) of the ML2 traces with different L/S values was measured using a vector network analyzer.

**Figure 12:**
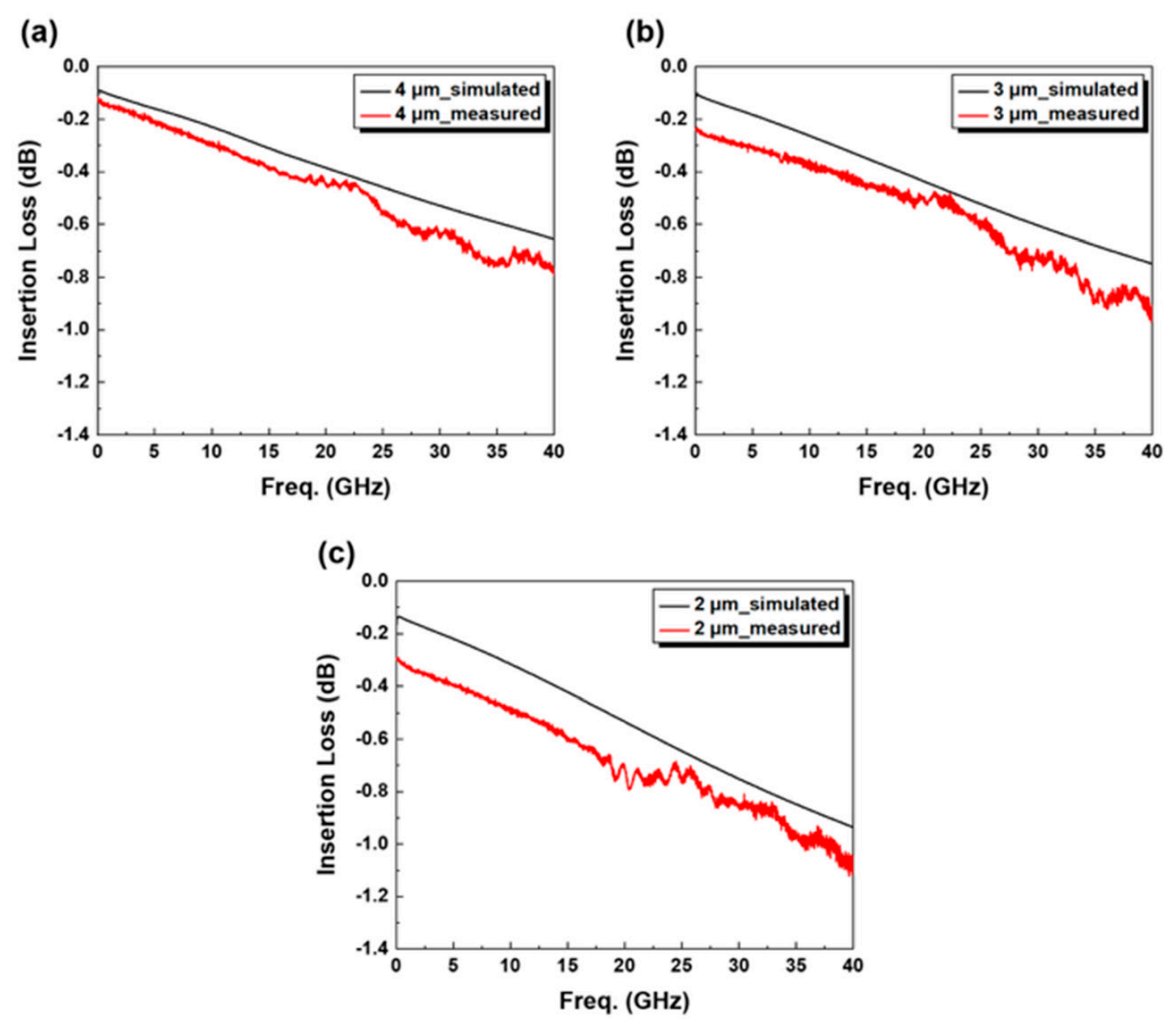
Insertion loss of the panel-level RDL interposer: (**a**) L/S = 4 μm/4 μm, (**b**) L/S = 3 μm/3 μm, and (**c**) L/S = 2 μm/2 μm.

As illustrated in Figure 12, the magnitude of the insertion loss increased with decreasing L/S, which is consistent with the simulation trend. Nevertheless, the insertion loss magnitude remained below 1.4 dB over 0–40 GHz, indicating that the interposer maintained low-loss characteristics. Electrical comparisons with the untreated, TMAC-treated, and CTAC-treated conditions were not performed because structurally equivalent fine-patterned test vehicles could not be established over the same L/S range. The untreated DFR exhibited pattern collapse at L/S = 2 μm/2 μm and 3 μm/3 μm, while the TMAC- and CTAC-treated DFRs exhibited pattern collapse across all evaluated dimensions. In contrast, BHAC-treated DFR remained stable over the entire evaluated range. In addition, the rinse treatment serves as a temporary photolithographic process, and the DFR is subsequently removed during RDL fabrication. Therefore, the electrical evaluation in this study was intended to verify the feasibility of the RDL interposer fabricated using the BHAC-enabled fine patterning rather than to isolate an intrinsic electrical effect of the rinse. Accordingly, the measured insertion loss of the test vehicle fabricated using the BHAC-containing rinse was compared with the 3D EM simulation results. Based on these results, the applicability of a BHAC-containing rinse was demonstrated through the fabrication of a panel-level RDL interposer.

## 4. Conclusions

This study systematically investigated the effects of cation-containing rinses on the surface chemistry, mechanical properties, polymer networks, and pattern collapse behavior of DFR. Although TMAC, CTAC, and BHAC decreased the SFE and increased the surface hydrophobicity, different patterning results were obtained. Pattern collapse worsened after TMAC or CTAC treatment owing to degradation of the mechanical properties, whereas BHAC treatment effectively prevented pattern collapse because the mechanical strength was enhanced.

Confocal Raman microscopy confirmed the diffusion of cations into the DFR. FTIR spectroscopy and swelling tests showed that TMAC and CTAC acted as plasticizers, loosening the polymer network and decreasing the apparent crosslink density. In contrast, BHAC treatment enhanced hydrogen-bonding within the DFR and resulted in a higher apparent crosslink density, consistent with the improved mechanical robustness.

Finally, the BHAC-containing rinse was selected as the optimal rinse and applied to fabricate a panel-level RDL interposer. Cu traces with a line/space of 2 µm/2 µm–4 µm/4 µm were successfully formed using a 7-µm-thick negative-tone DFR. The fabricated interposer exhibited an insertion loss magnitude below 1.4 dB over 0–40 GHz, demonstrating the applicability of the BHAC-containing rinse to a panel-level RDL interposer fabrication.

These results indicate that pattern collapse can be effectively prevented by simultaneously reducing the water–DFR interaction through hydrophobic surface modification and maintaining sufficient mechanical robustness through BHAC–DFR intermolecular interactions. In future work, we will evaluate the applicability of the rinse treatment to laser direct imaging (LDI), projection lithography, and electron-beam lithography, as well as the long-term stability of the hydrophobic surface modification. In addition, the mechanical and polymer-network changes of LPR and DFR by cationic rinse treatment will be systematically compared to further clarify the different behaviors of the two photoresist systems.

## 5. Patents

The patent resulting from the work reported in this manuscript is listed below: “Rinsing solution for photolithography and method for forming photosensitive resin pattern using the same”, Korean Patent No. 10-2932495.

## Figures and Tables

**Table 1:** Chemical structures of cations used in rinses A–C.

Rinse	Cation
A	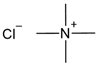
B	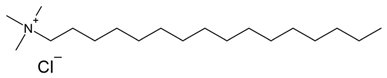
C *	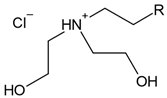

* Korean Patent No. 10-2932495.

**Table 2:** Formulation of rinses A–C.

Rinse	Cation	Concentration (mM)	pH
A	TMAC	10	7.0
B	CTAC	10	7.0
C	BHAC	10	7.0

**Table 3:** Surface chemical properties of standard liquids.

i	Standard Liquid	$\gamma_{\mathrm{Li}}$ (mN/m)	$\gamma_{\mathrm{Li}}^{d}$ (mN/m)	$\gamma_{\mathrm{Li}}^{p}$ (mN/m)
1	Deionized Water (DIW)	72.8	21.8	51.0
2	Ethylene Glycol (EG)	48.0	29.0	19.0
3	n-Hexane (Hex)	18.4	18.4	0

**Table 4:** Surface chemical properties of DFR after rinse treatment.

Rinse (Cation)	Treatment Time (s)	θ_DIW_ (°)	θ_EG_ (°)	θ_Hex_ (°)	Dispersive Component (mN/m)	Polar Component (mN/m)	Surface Free Energy (mN/m)	Interfacial Free Energy (mN/m)	Work of Adhesion (mN/m)
None	0	40.37	40.07	10.12	14.56	37.97	52.53	1.69	123.64
A (TMAC)	20	42.95	44.72	10.89	14.24	36.12	50.36	2.08	121.07
	40	46.32	43.52	10.64	14.81	33.69	48.50	2.46	118.84
	50	44.95	43.74	10.82	14.60	34.69	49.30	2.28	119.81
	60	46.35	44.42	10.65	14.70	33.65	48.35	2.50	118.65
B (CTAC)	20	46.75	44.88	9.97	14.72	33.32	48.04	2.57	118.27
	40	51.49	49.88	9.35	14.68	29.71	44.38	3.56	113.62
	50	50.93	47.96	10.26	14.83	30.19	45.03	3.38	114.44
	60	51.44	50.27	9.81	14.59	29.75	44.35	3.57	113.58
C (BHAC)	20	52.12	40.16	10.08	16.04	29.41	45.46	3.39	114.86
	40	60.33	41.03	10.15	17.18	23.32	40.50	5.63	107.68
	50	60.22	40.59	10.08	17.23	23.40	40.63	5.58	107.85
	60	60.52	40.53	10.11	17.28	23.18	40.46	5.68	107.58

**Table 5:** Parameters of the swelling tests.

Rinse (Cation)	V_s_ (L/mol)	ρ_p_ (g/mL)	ρ_s_ (g/mL)	γ (mN/m)	δ_p_ (MPa^0.5^)	δ_s_ (MPa^0.5^)	χ	Swelling Ratio (%)	Crosslink Density (mol/L)
None	0.0766	1.25	0.785	52.5	26.6	23.5	0.293	125	15.5
A (TMAC)	0.0766	1.25	0.785	48.5	25.5	23.5	0.129	132	14.3
B (CTAC)	0.0766	1.25	0.785	44.4	24.4	23.5	0.0275	143	11.8
C (BHAC)	0.0766	1.25	0.785	40.5	23.3	23.5	0.000747	127	18.6

**Table 6:** Design rule of the panel-level RDL interposer.

	Elements	Thickness (μm)	L/S (μm/μm)	BMV Diameter (μm)
RDL 1	ML1	20	Ground Plane	
	DL1	10		10
RDL 2	ML2	2	2/2, 3/3, 4/4	
	DL2	10		10
RDL 3	ML3	3	5/20, 20/20, 75/20	

## Data Availability

The original contributions presented in this study are included in the article. Further inquiries can be directed to the corresponding authors.
